# Estimating Annual Soil Carbon Loss in Agricultural Peatland Soils Using a Nitrogen Budget Approach

**DOI:** 10.1371/journal.pone.0121432

**Published:** 2015-03-30

**Authors:** Emilie R. Kirk, Chris van Kessel, William R. Horwath, Bruce A. Linquist

**Affiliations:** 1 Department of Plant Sciences, University of California Davis, Davis, California, United States of America; 2 Department of Land, Air, and Water Resources, University of California Davis, Davis, California, United States of America; University of Maryland, UNITED STATES

## Abstract

Around the world, peatland degradation and soil subsidence is occurring where these soils have been converted to agriculture. Since initial drainage in the mid-1800s, continuous farming of such soils in the California Sacramento-San Joaquin Delta (the Delta) has led to subsidence of up to 8 meters in places, primarily due to soil organic matter (SOM) oxidation and physical compaction. Rice (*Oryza sativa*) production has been proposed as an alternative cropping system to limit SOM oxidation. Preliminary research on these soils revealed high N uptake by rice in N fertilizer omission plots, which we hypothesized was the result of SOM oxidation releasing N. Testing this hypothesis, we developed a novel N budgeting approach to assess annual soil C and N loss based on plant N uptake and fallow season N mineralization. Through field experiments examining N dynamics during growing season and winter fallow periods, a complete annual N budget was developed. Soil C loss was calculated from SOM-N mineralization using the soil C:N ratio. Surface water and crop residue were negligible in the total N uptake budget (3 – 4 % combined). Shallow groundwater contributed 24 – 33 %, likely representing subsurface SOM-N mineralization. Assuming 6 and 25 kg N ha-1 from atmospheric deposition and biological N2 fixation, respectively, our results suggest 77 – 81 % of plant N uptake (129 – 149 kg N ha^-1^) was supplied by SOM mineralization. Considering a range of N uptake efficiency from 50 – 70 %, estimated net C loss ranged from 1149 – 2473 kg C ha^-1^. These findings suggest that rice systems, as currently managed, reduce the rate of C loss from organic delta soils relative to other agricultural practices.

## Introduction

Pressure from burgeoning population and increasing demand for agricultural production has affected nearly every biome on Earth, and human activity has resulted in detrimental effects in many sensitive ecosystems including important river deltas [1] and peatlands [2]. Peatlands are landscapes characterized by organic soils at least 30–40 cm thick (e.g. Histosols, peat, muck; generally ≥12% organic carbon (C)) that form over millennia from accumulated biotic materials under conditions of slow decomposition, such as cold temperatures and or prolonged inundation [3, 4, 5]. Because of the unique conditions under which they form, peatlands are sensitive ecosystems highly susceptible to disturbance from climate change and from human use [6, 7]. While peatlands occupy only 3% of the global terrestrial area [8], their soils contain >25% of the global soil C stocks [4], making disturbed peatlands a significant source of greenhouse gas (GHG) emissions [6, 9, 10, 11, 12]. When organic soils are drained, the land surface subsides due to soil compaction and as a direct result of SOM oxidation and gaseous C losses, with the relative importance of oxidation increasing as the main driver of continued subsidence over time [13, 14, 15, 16].

Despite this potential for severe degradation, 14–20% of peatlands are used for agricultural production worldwide [8]. Subsidence in agricultural peatlands has been reported in New Zealand [17], Southeast Asia [18, 19, 20], Florida, USA [21, 22], Northern Europe [23], and in the Sacramento-San Joaquin Delta, California, USA (hereafter the Delta) among others. Since first being drained in the mid-1800s, the organic soils found in the central part of the Delta have been used primarily for maize (*Zea mays*), forage, and vegetable production, and have subsided as much as 8 m in many places [13, 24]. Current rates of subsidence in the Delta are generally between 1–3 cm yr^-1^ and can largely be attributed to microbial oxidation of SOM after soils are permanently drained with further consolidation of the remaining mineral soil concurrent to this oxidation [13, 16, 25].

Cultivation of irrigated paddy rice (*Oryza sativa*) in the Delta has recently been found to slow soil subsidence—more closely mimicking the naturally flooded state of the peat—relative to currently dominant upland crops such as maize [26]. Anaerobic conditions in the flooded soil inhibit SOM decomposition [27]. In addition, temperature buffering effects of flooded soils are particularly valuable during summer, the period of highest potential microbial respiration [16, 28]. However, observations from a rice N fertilizer study on these soils found that N uptake exceeded 200 kg N ha^-1^ in N fertilizer omission plots [29], a rate of uptake >30% higher than average N uptake values in fertilized plots for typical California rice [30]. Therefore, we hypothesized that the majority of N uptake was derived from SOM, meaning that mineralization and loss of soil C and N is still occurring under flooded rice production in the Delta.

This study was designed to assess an N budget approach for estimating soil C loss rates of peatland soils under flooded rice production. Experiments were designed to isolate and quantify the different sources of N supporting plant uptake—namely SOM, irrigation water, shallow groundwater, and crop residue—to determine the amount of N mineralization from the soil. In turn, net soil C loss was estimated based on the soil C:N ratio and adjusted for C inputs from crop residues.

## Materials and Methods

### Site characteristics and rice system management

We conducted a study to determine sources of plant available N on Twitchell Island, CA, in the western part of the Delta (latitude: 38.106°N, longitude: 121.655°W). The study site is owned by the California Department of Water Resources and leased to farmers for agricultural production. California Department of Water Resources allowed the use of the rice fields on Twitchell Island for this study as well. Twitchell Island currently lies as much as 6 m below sea level [13] and is protected on all sides by artificial levees. The climate is Mediterranean with mild, wet winters and dry summers. From 1998–2013 the mean annual temperature was 9°C minimum and 22°C maximum [31]. Soils at the site are classified as Euic, thermic, Typic Haplosaprists (Rindge mucky silt loam, 0–2% slope) [32]. Total soil C in the experimental sites ranged from 129–154 g kg^-1^ (Table 1).

**Table 1:** Summary of soil characteristics for Twitchell Island rice fields.

Site	pH	Total C (g kg^-1^)	Total N (g kg^-1^)	C:N	Unfertilized N uptake (kg N ha^-1^)	Total P (g kg^-1^)	Total K (g kg^-1^)
1	5.50	154	10.7	14.4	185	1.0	1.2
2	6.01	129	8.7	14.8	167	1.1	1.2

Soil pH, total P, and total K values are based on composite samples from 0–15cm depth; total C, total N, and C:N values are based on composite samples from 0–30 cm. Unfertilized N uptake is the average of 4 replicates at each site, and is the total amount of N in aboveground biomass (straw + grain) at crop maturity.

In the Delta, rice is typically drill seeded from mid-April to mid-May each season. Due to the relatively cool temperatures compared to the main rice growing region in California’s Sacramento Valley, short duration rice varieties are grown. Fields are flooded approximately 1 month after planting and kept flooded until August/September when they are drained in preparation for harvest. Following harvest the rice residue is chopped and left on the soil surface over the winter fallow season. To facilitate straw decomposition [33] and provide wildfowl habitat, fields are flooded again from November through February before being drained for land preparation the following spring.

### Annual N budget and soil C loss model overview

**Fig 1:**
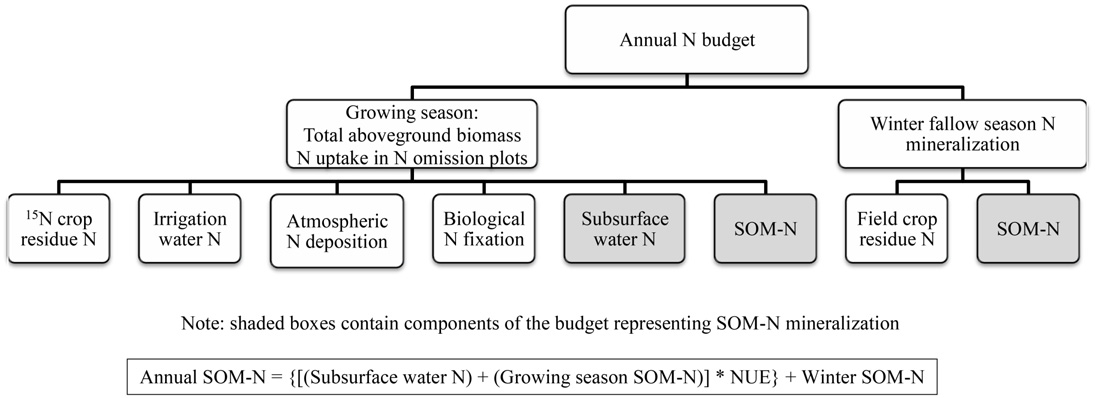
Conceptual model of the annual (growing and winter fallow season) N budget. The growing season portion is based on aboveground biomass N uptake in 0N plots and N uptake contributions from crop residue, water, SOM sources, atmospheric N deposition, and biological N fixation. The winter fallow season N budget is comprised of crop residue and SOM mineralization. All components of the model were determined experimentally except the values for atmospheric N deposition and biological N fixation which were estimated from available literature.

An annual N budget was constructed to estimate the amount SOM-N mineralization during the growing and winter fallow seasons (Fig 1). Total mineralization was used to estimate annual soil C loss under rice cultivation. The total annual N budget was comprised of SOM-N mineralized during the growing (May—October) and winter fallow (November—April) seasons. Growing season N was determined based on N uptake of total aboveground biomass in N fertilizer omission plots. Several experiments were conducted to determine the contribution of different environmental sources to N uptake. Annual SOM-N mineralization was the sum of growing season SOM-N, and the winter fallow SOM-N mineralization. The soil C:N ratio was used to calculate an annual soil C loss based on the total annual SOM-N mineralized, and net soil C loss was determined by accounting for C inputs from crop residues.

### Growing season N budget

Field experiments were conducted during the 2012 growing season at two sites. Throughout the experimental period, the rice variety M-104 was grown and the fields were managed by the farmer following normal rice cultivation practices as described earlier. One difference between Site 1 and 2 was an early season irrigation flush lasting approximately seven days at Site 1 that did not occur at Site 2.

#### N uptake in fertilizer N omission plots

Baseline N uptake was determined from four fertilizer N omission plots (4 X 4 m) at each site. Phosphorous (P) and potassium (K) fertilizer were applied to these plots at 50 kg P_2_O_5_ ha^-1^ and 100 kg K_2_O ha^-1^ to ensure these nutrients were not limiting plant growth. For determination of aboveground biomass and grain yield, the crop was harvested at physiological maturity by cutting at ground level from a 1.1 to 1.2 m^2^ area. After weighing, subsamples were collected, weighed, and dried to a constant weight at 60°C. Grain and straw fractions were separated, ground and analyzed for N content.

#### N derived from residue

The amount of plant N uptake during the 2012 growing season contributed by the previous year’s crop residue was determined using ^15^N-labeled residue incorporated into the soil in spring 2012. ^15^N-enriched rice residue was generated by growing rice (cv. M-104) over winter 2011–2012 in a greenhouse where plants were fertilized with 10 atom % ^15^N-enriched (NH_4_)_2_SO_4_. Aboveground biomass was harvested when the plants reached maturity. The grains were removed and the residue was cut into 5–6 cm long pieces to mimic straw chopping in the field. The residue was partially decomposed in deionized water inoculated with Twitchell Island field soil, receiving the same total heat units (base temp of 8°C) during decomposition as the corresponding field residue to ensure the ^15^N-labeled residue approximated the residue that remained on the soil surface under field conditions over the winter fallow. After partial decomposition, the ^15^N-labeled residue was rinsed with deionized water and air-dried for ease of handling prior to incorporation in the field. The ^15^N enrichment of labeled rice residue was 9.0693 atom %, and the C:N ratio of the ^15^N-labeled residue was 41, similar to the field residue which had a C:N of 35 (Table 2). Before incorporation in the field, ^15^N-labeled residue was mixed with unlabeled field residue to generate enough total material equivalent to residues remaining in the field after normal winter decomposition. The total application rate was 1310 kg ^15^N-labeled residue ha^-1^ and 3700 kg unlabeled field residue ha^-1^. At the beginning of the 2012 rice season, field residues were removed from the experimental sites by manually raking before tillage. Microplots (1 m^2^) were delineated and the ^15^N residue mix was incorporated at a depth of 20 cm after tillage and prior to planting. No-residue, ^15^N-residue, and ^15^N-residue + ^14^N-fertilizer (80 kg N ha^-1^ urea) treatments were included, replicated four times in a randomized complete block design (RCBD) at each site. Fertilizer P and K were applied to all treatments at 50 kg P_2_O_5_ ha^-1^ and 100 kg K_2_O ha^-1^ to ensure these nutrients did not limit plant growth.

**Table 2:** Characteristics of ^15^N-labeled rice residue applied to microplots.

	^15^N residue	^14^N field residue
Dry weight residue added, kg ha^-1^	1310	3700
Total N, kg ha^-1^	12	35
C:N ratio of residue	41	35
^15^N content (atom %)	9.0693	0.3673

Residue mixture was incorporated manually prior to planting in 2012.

At the end of the season, ten tillers were harvested from the center of each microplot for ^15^N enrichment measurements. Aboveground biomass yield samples were harvested from a 0.64 m^2^ area in the center of each microplot and processed as described above. Four replicate 10-tiller samples were also collected from the area >5 m away from the residue treatment plots in each field to determine background ^15^N in the crop. ^15^N recovery was determined in the rice aboveground biomass and soil to 30 cm, and total recovery was calculated. After harvest, atom % ^15^N values were used to calculate the relative contribution to total N uptake from the labeled residue (fNdr) using the following equation:

$$fNdr\;=\;(atom{\%}^{15}N_{sample}–\;atom{\%}^{15}N_{background})\;/\;(atom{\%}^{15}N_{input\;residue}–\;atom{\%}^{15}N_{background})$$ (1)

where atom % ^15^N values here are based on the enriched and background tiller samples collected at harvest and the input residue.

The overall amount of N derived from residue (Ndr) was calculated as follows:

$$Ndr\;=\;\left(fNdr\;*\;total\;AGB\;N\;uptake\right)\;*\;(proportion^{15}N\;residue\;applied)$$ (2)

where total AGB N uptake (kg N ha^-1^) is the aboveground biomass N uptake from the ^15^N-residue or ^15^N-residue + ^14^N-fertilizer treatment, and proportion ^15^N residue applied is the proportion of the total residue mixture applied that was comprised of ^15^N-labeled residue (g ^15^N residue applied / g residue mixture applied).

The percent ^15^N recovery in the aboveground biomass and soil, and percent ^15^N loss from the system using the following equations:

$${\%}^{15}N\;recovered\;=\;\left(fNdr\;*\;total\;N\;in\;pool\right)\;/\;(amount^{15}N\;applied)$$ (3)

where total N in pool (kg N ha^-1^) is determined at harvest and amount ^15^N applied (kg N ha^-1^), is based on the straw incorporation in the spring and

$${\%}^{15}N\;loss\;=\;100\%\;–\;({\%}^{15}N\;recovered\;aboveground\;+\;{\%}^{15}N\;recovered\;belowground)$$ (4)

#### N derived from irrigation water

The amount of N from surface irrigation water (Ndsw; kg N ha^-1^) was determined based on sampling from each site at the inlet to the field five times throughout the flooded period during the growing season. Evapotranspiration (ETa) was estimated using a crop coefficient for rice of 1.06 [34] and cumulative evapotranspiration (ET) of 0.634 m, or 6.34*10^6^ L ha^-1^, from California Irrigation Management Information System (CIMIS) data for Twitchell Island during the period of permanent flood, 21 June—21 September 2012 [31]. Therefore Ndsw (kg N ha^-1^) was calculated as:

$$Ndsw\;=\;average\;N_{inlet}*\;ETa\;*\;\left(1\;kg/10^{6}mg\right)$$ (5)

where average N _inlet_ (mg N L^-1^) is the seasonal average NH_4_-N and NO_3_-N concentration in the incoming irrigation water, and the ETa (L ha^-1^) is the cumulative ET adjusted for the crop coefficient.

#### N derived from shallow groundwater

An *in situ* mesocosm experiment was conducted to assess the N contribution from shallow groundwater upwelling to the total crop N uptake budget. Rice was grown in the field in 61 cm x 47 cm x 40 cm rectangular plastic mesocosms. After tillage, the mesocosms were installed by burying them in the soil such that the upper rim was level with the soil surface. Soil was removed to install the mesocosms, and then the soil was replaced by depth within each mesocosm. Mesocosms were seeded at the same time as the rest of the field. Two treatments (+ groundwater and—groundwater) were replicated four times in an RCBD design at Sites 1 and 2. The treatments receiving shallow groundwater (+groundwater treatment) had 3.5 cm diameter holes drilled in the base to remove 23% of the base area and facilitate water movement between the interior and surrounding subsoil. Treatments without holes excluded shallow groundwater (–groundwater treatment). Surface irrigation water was able to move freely across the surface of all mesocosms. Because surface water is moving uniformly across the surface of each mesocosm treatment, there is no net effect of surface irrigation water N on the assessment of groundwater N contribution. Fertilizer P and K (50 kg P_2_O_5_ ha^-1^ and 100 kg K_2_O ha^-1^) were applied to ensure these nutrients were not limiting plant growth; no N fertilizer was applied. Ten cm long Rhizon MOM porewater samplers (RRP, Wageningen, Netherlands) were installed at 20 cm depth inside each + groundwater and—groundwater treatments, and additionally at 45 cm depth outside the—groundwater treatment to measure porewater NO_3_-N and NH_4_-N throughout the growing season. Soil porewater was sampled six times during the growing season when fields were flooded. Porewater samples were collected using evacuated Exetainer vials acidified with 0.25 mL 1.0 M H_2_SO_4_ to pH ≤2.

Aboveground biomass was harvested and processed as described in the previous experiments, except the entire area of each mesocosm was harvested. The amount of N from shallow groundwater (Ndgw; kg N ha^-1^) was calculated using the following equation:

$$Ndgw\;=\;{\left(N\;uptake\right)}_{+}{}_{groundwater}–\;{\left(N\;uptake\right)}_{-}{}_{groundwater}$$ (6)

where N uptake (kg N ha^-1^) is total aboveground biomass N uptake in each treatment at harvest.

Deep groundwater samples were collected to provide additional detail on N pools in the system. Deep groundwater sampling from wells maintained by HydroFocus Inc. (Davis, CA) was conducted on 7–8 August 2012 using HydroFocus standard operating procedures. Samples were stored on ice and in the dark during transport. Groundwater samples for NH_4_-N analysis were acidified with 1 M H_2_SO_4_ to pH ≤2 in the lab. All water samples were filtered to a mesh size of 0.45 μm. Samples were then frozen until analysis.

#### N derived from growing season SOM mineralization

The amount of N from peat mineralization (Ndp _growing_; kg N ha^-1^) was calculated as the difference remaining between total N uptake from the N omission plots (0N uptake) and N attributed to other known sources:

$$Ndp_{growing}=\;0N\;uptake\;–\;(Ndr\;+\;Ndsw\;+\;Ndgw\;+\;N_{deposition}+\;N_{fixation})$$ (7)

with all values measured in kg N ha^-1^. The values for 0N uptake, Ndr, Ndsw, and Ndgw were determined in the experiments outlined previously, while two other potential sources of N in this system—atmospheric N deposition (N _deposition_) and biological N_2_ fixation (N _fixation_)—were estimated based on available literature. While these potential environmental sources of N have yet to be studied in rice systems in the Delta, recent regional estimates for total annual atmospheric N deposition in the central valley are approximately 6 kg N ha^-1^ yr^-1^ including both wet and dry deposition [35]. The full 6 kg N ha^-1^ was accounted for in the growing season N budget in this study. Also, biological N_2_ fixation by free-living cyanobacteria in rice paddies generally contributes 20–30 kg N ha^-1^ yr^-1^ to the annual N budget [36, 37, 38, 39]. For this study, an intermediate value of 25 kg N ha^-1^ was included in the growing season N budget for this study. While this is likely an overestimate as N availability is not limiting in this system to drive high levels of N_2_ fixation, including this component in the N budget makes the estimated total subsidence more conservative.

This Ndp represents the SOM-N that was actually taken up by the plants; however, plants are not 100% efficient in taking up nutrients so it is necessary to adjust the Ndp to account for N uptake efficiency (NUE). The NUE used for this estimate is 50%, a commonly reported value for fertilizer-N in rice [40, 41, 42]. The N losses from the system, including potential gaseous losses and leaching, are combined in the NUE term and not specifically estimated in this study. To assess the impact of NUE on the estimated soil C loss, a sensitivity analysis was conducted. The total growing season SOM-N mineralization (N min _growing_; kg N ha^-1^) was calculated as:

$$N\;min_{growing}=\;Ndp_{growing}/\;NUE$$ (8)

### Winter fallow season N budget

#### N mineralized from residue

For determining the amount of N mineralized from field residue during the winter fallow season, residue samples were collected in fall 2011 and again in spring 2012 to quantify the change in N content of residues during this period. Residue samples were oven dried and prepared for analysis as described earlier for aboveground biomass samples. These samples were used to determine pre- and post-decomposition total C and N in the residue. Estimates of winter decomposition of the rice residue were based on 2011 harvest yield data and spring residue sampling in 8 m^2^ plots with five replicate samples from each site. Spring samples were collected from the experimental site where all field residue was subsequently removed for the ^15^N-labeled residue experiment. Residue was weighed in the field and subsamples were taken for analysis. The amount of N mineralized from residue overwinter (kg N ha^-1^) was then estimated using the following equation:

$$N\;mineralized\;from\;residue\;=\;residue\;N_{fall}–\;residue\;N_{spring}$$ (9)

where residue N _fall_ (kg N ha^-1^)was the total N in residue based on 2011 harvest data and sampled residue N concentrations, and residue N _spring_ (kg N ha^-1^)was based on sampled plots residue mass and N concentration.

#### N mineralized from SOM

To determine mineralization of SOM-N during the winter fallow season soil samples were collected from each site prior to planting and again prior to the summer flood establishment in 2012, and analyzed for NO_3_-N and NH_4_-N accumulation. Soil samples were collected as the composite of five cores to 15 cm from a transect across each block in each experiment at both Site 1 and 2. Bulk density was sampled for each block to 15 cm using plastic cylinders. Samples were transported on ice to the lab for extraction of NH_4_-N and NO_3_-N within 48 hours.

Soil NO_3_-N represents accumulated N mineralized from SOM and the crop residue; therefore, after subtracting the N mineralized from crop residue the remainder may be attributed to SOM decomposition. The fallow season SOM-N mineralization (N min _fallow_; kg N ha^-1^) was calculated using the following equation:

$$N\;min_{fallow}=\;soil\;N0_{3}-N\;at\;flooding\;–\;residue\;N\;mineralized\;overwinter$$ (10)

where all values are calculated in kg N ha^-1^.

### Estimating soil C loss based on SOM-N mineralization

Annual SOM-N mineralization (N min _total_; kg N ha^-1^) was calculated using the following equation:

$$N\;min_{total}=\;N\;min_{growing}+\;N\;min_{fallow}$$ (11)

where all values are calculated in kg N ha^-1^. Soil C loss was estimated using soil C:N ratio. The soil C and N values were based on composite samples of five cores collected with an auger (diameter: 1.9 cm) to 30 cm with 12 replicate samples per site. Soils were dried overnight at 65°C, weighed, homogenized, and pulverized using a ball mill before analysis.

Based on the C:N ratio of the soil (kg C kg^-1^ N), a total mass of C lost (kg C ha^-1^) was estimated using the values obtained for annual N mineralization (N min _total_; kg N ha^-1^). The C input from crop residue at tillage (kg C ha^-1^) was added to assess the net C loss (kg C ha^-1^). Annual net C lost was calculated using the following equation:

$$Net\;C\;loss\;=\;(N\;min_{total}*\;C:N\;ratio)\;+\;C\;input\;from\;residue$$ (12)

### Soil, water, and plant analysis

Soils were analyzed for extractable mineral N using a cold 2 M KCl extraction within 48 h of sampling. Mineral N content was determined using colorimetric methods for NO_3_-N [43, 44] and NH_4_-N [45, 46] analyzed on a Shimadzu UV-160 spectrophotometer (Shimadzu Inc., Tokyo, Japan). The remainder of each sample was air-dried and saved for further analysis. Soil pH, total P, and total K were analyzed at the UC Davis DANR lab, and total C and N were analyzed at the UC Davis Stable Isotope Facility. Soils sampled from the ^15^N-labeled residue experiment at harvest to 30 cm were sent to the UC Davis Stable Isotope Facility for ^15^N, total N, and total C analysis using an Elementar Vario EL Cube or Micro Cube elemental analyzer (Elementar Analysensysteme GmbH, Hanau, Germany) interfaced to a PDZ Europa 20–20 isotope ratio mass spectrometer (Sercon Ltd., Cheshire, UK).

Water samples were analyzed using the colorimetric method for extractable NO_3_-N [43, 44] and NH_4_-N [45, 46].

Plant tissue total C, total N, and atom % ^15^N analysis was carried out by the UC Davis Stable Isotope Facility (Davis, CA) using a PDZ Europa ANCA-GSL elemental analyzer interfaced to a PDZ Europa 20–20 isotope ratio mass spectrometer (Sercon Ltd., Cheshire, UK).

### Data analysis

Statistical analysis was completed in SAS (SAS Institute Inc. 2010), using a general linear model procedure command to run analysis of variance for fertilizer, water, and ^15^N-residue treatment effects on rice grain yield and aboveground biomass N uptake. The significance level was set a priori to *p* <0.05. Mean comparisons were made using the LSMeans statement and Tukey’s honest significant difference. Standard errors were calculated based on direct measurements, and are based on the appropriate propagation of error formulae where parameters are not associated directly with replicated measurements, such as data aggregated by site. For calculating the standard error of sums or differences (*SEz*), the following equation was used:

$$\;SEz=\;\surd ({\left(SEa\right)}^{2}+\;{\left(SEb\right)}^{2}+\;{\left(SEc\right)}^{2}\ldots )$$ (13)

where SEa, SEb, SEc, and so on are the standard errors of each measured value. For conversion factors multiplying or dividing by an exact number, the standard error is multiplied by the same conversion factor [47].

## Results and Discussion

### Growing season N uptake fertilizer N omission plots

The total aboveground plant N uptake from the fertilizer N omission plots ranged from 167 kg N ha^-1^ (Site 2) to 185 kg N ha^-1^ (Site 1) and formed the baseline for the growing season N budget (Table 1). These values are in line with uptake observed in N omission plots from 2011 at the same site [29]; however they are high relative to other reports from California rice systems where rice is grown on mineral soils. For example, in one study N uptake in N omission plots ranged from 45–90 kg N ha^-1^[30], which is more than 50% lower than the average we observed.

Grain yield in the N omission plots was also high: 7.9 Mg ha^-1^ (Site 1) and 10.7 Mg ha^-1^ (Site 2). The lower yield at Site 1 was likely due to higher weed pressure; however Site 2 yields were well above the California state average of 9.3 Mg ha^-1^ [48].

### Plant N uptake from ^15^N-labeled residue

**Fig 2:**
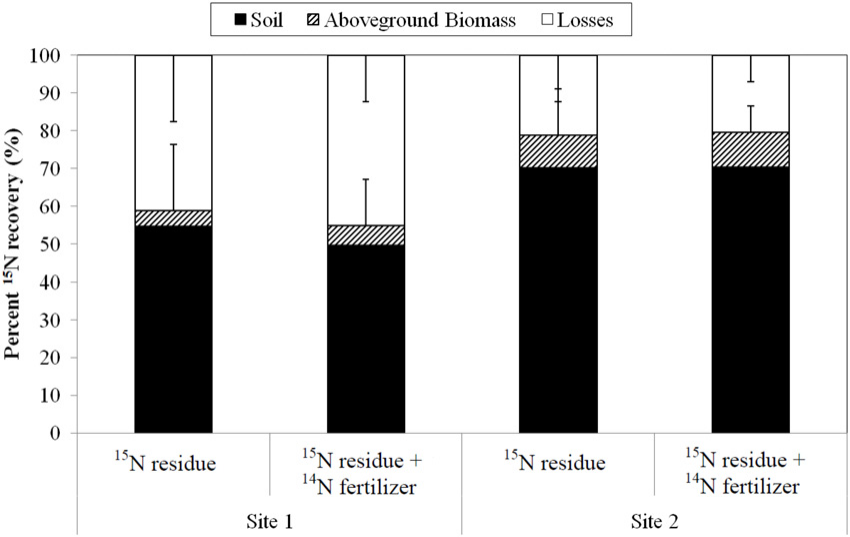
The fate of ^15^N from labeled rice residue at harvest from two treatments: addition of ^15^N-labeled residue without fertilizer (^15^N-residue), and addition of ^15^N-labeled residue with 80 kg N ha^-1 14^N-urea (^15^N-residue + ^14^N-fertilizer). Error bars are the standard error of the mean losses or total recovery (n = 4).

Total aboveground N uptake in the ^15^N-labeled residue treatment without N fertilizer was 135 kg N ha^-1^ (Site 1) and 128 kg N ha^-1^ (Site 2) (Table 3). The lower N uptake in these plots compared to the N omission plots described previously could be due to increased N immobilization driven by the crop residue treatments in this experiment [49]. Based on final ^15^N enrichment at harvest, results indicate less than 3% of the rice N uptake was supplied by the prior year’s crop residue: only 1.9 kg N ha^-1^ (Site 1) and 3.8 kg N ha^-1^ (Site 2) (Table 4, S7 Table). Of the ^15^N-labeled residue, 50–70% was recovered in the soil, similar to other studies [50, 51], suggesting it will potentially be available to subsequent crops and subject to other losses (Fig 2). The contribution to N uptake from the ^15^N-labeled residue in this study is conservative due to losses that occurred during the partial decomposition prior to incorporation, but the value is still in agreement with earlier studies which show limited contribution from residue in the first year following incorporation of the residues [50, 51]. At each site in this study, when no residue was applied, there was no significant difference in total N uptake compared to the total N in the crop when ^15^N-residue was applied (*P*>0.05; Table 3), suggesting immobilization occurs when rice residue is applied [49]. The intensity of immobilization of soil N is controlled by the chemical characteristics of rice crop residues [52, 53], and by an increase in soil microbial biomass stimulated by the addition of fresh, labile residue-C [49, 54]. These observations are relevant for N availability during the growing season, even as N immobilization in flooded soils is lower compared to aerobic soils due to reduced microbial activity [55]. The high level of indigenous N supply as shown by the high crop N uptake in the no-residue treatment (Table 3) and the apparent immobilization of ^15^N-residue supports the conclusion that residue N only contributes a minor amount to the subsequent rice crop. However, considering the cumulative effect of incorporating residues over multiple growing seasons has been found to supply close to 20 kg N ha^-1^ to the crop [33, 56]. Site 1 had higher crop N uptake than Site 2 across all treatments (Table 3), possibly due to an additional wet-dry cycle that stimulated residue mineralization early in the season at this site [57, 58, 59].

**Table 3:** Total aboveground biomass (straw + grain) N uptake in ^15^N-labeled residue experiment.

	Aboveground biomass N uptake	
Treatment	Site 1 (kg N ha^-1^)	Site 2 (kg N ha^-1^)
No residue	168 ab	138 a
^15^N residue	135 b	128 a
^15^N residue + ^14^N fertilizer	190 a	175 a

Within each site, uptake values followed by the same letter are not significantly different (p<0.05).

**Table 4:** Estimated growing season mineralization based on plant uptake.

Estimated growing season mineralization	Site 1		Site 2	
	(kg N ha^-1^)	SE	(kg N ha^-1^)	SE
Total N uptake	185.0	10.0	167.0	7.7
*N derived from*:				
Residue^a^	1.9	0.2	3.8	1.1
Surface water^b^	2.9	1.3	3.0	1.3
N deposition^c^	6		6	
Biological N_2_ fixation^d^	25		25	
Peat (total)	149.2	16.2	129.2	12.3
Subsurface sources^e^	61.7	9.0	39.6	6.7
Surface peat^f^	87.5	13.5	89.6	10.3
Growing season peat N mineralization^g^	298.4	32.5	258.3	24.7

Total N uptake is based on aboveground biomass (straw + grain) from plots receiving no N fertilizer. Standard errors (SE) are calculated using standard propagation of error formulae.

^a^ based on ^15^N-labeled residue tracer study

^b^ based on water sampling and ETa

^c^ [35]

^d^ [36, 37, 38]

^e^ based on N uptake differences between mesocosm treatments. Shallow groundwater N assumed to be from peat

^f^ calculated as the difference between total N uptake and the N derived from other sources

^g^ Growing season peat mineralization is the total N derived from peat adjusted to account for an NUE of 50%

### Plant N uptake from water sources

Surface water samples collected during the growing season had levels ≤0.7 mg L^-1^ NH_4_-N and ≤0.3 mg L^-1^ NO_3_-N at all sampling times, making irrigation water a negligible source of N for the crop (S1 Table). The low N concentrations in the surface water also suggest that the N attributed to shallow groundwater was not from surface water that percolated into the subsurface. Using the inlet surface water N concentrations multiplied by ETa (Eq 5), it was estimated that irrigation water contributed 2.9 kg N ha^-1^ (Site 1) and 3.0 kg N ha^-1^ (Site 2) (Table 4,S7 Table).

In contrast, shallow groundwater provided 40–60 kg N ha^-1^ toward total plant N uptake. Total aboveground N uptake in the + groundwater treatment was 122 (Site 2) to 188 (Site 1) kg N ha^-1^ which was significantly higher than in the—groundwater treatment which was 83 (Site 2) to 127 (Site 1) kg N ha^-1^(S2 Table). This finding was unexpected and shows that upward movement of previously mineralized N in the shallow groundwater may contribute a substantial amount to total crop N demands in this system. Porewater samples from inside the mesocosms yielded low N concentrations throughout the season (≤0.2 mg N L^-1^), reflecting active and continual crop uptake. The natural abundance ^15^N signature of the rice plants suggests that the primary source of N was the same in the presence or absence of shallow groundwater: 0.3685 atom % ^15^N for both treatments at Site 1, and 0.3683–0.3685 atom % ^15^N in + groundwater and—groundwater treatments at Site 2. This indicates a 0.0024–0.0026 atom % ^15^N enrichment relative to urea (0.3659 atom % ^15^N; [60]), suggesting that N from below 40 cm is likely the result of SOM mineralization. Additionally, samples of groundwater from deeper wells at each site showed high levels of NH_4_-N up to 18.8 mg L^-1^ as deep as 5.2 m, and no detectable NO_3_-N (S3 Table). Because there was no accumulation of NO_3_-N at depth and because this system is characterized by alternating aerobic and anaerobic conditions, leaching is not evidenced and residual fertilizer from previous years was most likely denitrified and lost from the system [61, 62]. It is therefore reasonable to attribute these subsurface N sources to SOM mineralization.

### Plant N uptake from SOM-N mineralization

Combined N uptake from surface SOM and subsurface SOM was 129 kg N ha^-1^ (Site 2) and 149 kg N ha^-1^ (Site 1) (Table 4,S7 Table). These were adjusted for NUE which we assumed to be 50%. Thus the total estimate of SOM-N mineralized during the growing season was 258 kg N ha^-1^ (Site 2) and 298 kg N ha^-1^ (Sites 1) (Table 4,S7 Table). The estimated SOM-N mineralization and soil C loss is sensitive to the NUE assumption and will be discussed later.

### Winter fallow season SOM-N mineralization

Based on field residue samples collected in fall 2011 and spring 2012, the crop residue remaining at each site prior to spring tillage indicates that approximately 45% of the residue biomass decomposed during the fallow season (S4 Table). Similar values are reported elsewhere for the same residue management practices in California [33, 63]. Accounting for this rate of decomposition is important because it represents rapid C losses and reduces the annual net C balance compared to the total residue biomass input at harvest. Accounting for fallow season decomposition was also the impetus for the partial decomposition of the ^15^N-labeled residue prior to incorporation in the spring, bringing the C:N ratios of the two materials into close approximation (Table 2). Losses of C from residues during the fallow season exceeded N mineralization from the residue over the same period, where only an estimated 16.9 (Site 1) to 21.0 (Site 2) kg N ha^-1^ mineralized (Table 5,S7 Table). Soil samples from late spring immediately prior to flooding the fields showed NO_3_-N accumulation of 20.3 (Site 1) to 42.1 (Site 2) kg N ha^-1^ (Table 5,S7 Table), a pool of N that for the purposes of this model we assumed was denitrified and lost following flooding [61]. The lower soil NO_3_-N observed at Site 1 is likely due to an additional early season irrigation flush at this site which may have caused additional N losses. Denitrification and NH_3_ volatilization are the primary loss mechanisms, and leaching is generally minimal in rice systems [62, 64]. The fallow season N losses not accounted for in this model result in a lower estimate of winter SOM-N mineralization and consequently decrease the final soil C loss estimate. However, these possible uncertainties are minor as the vast majority of SOM-N mineralization occurred during the growing season (258–298 kg N ha^-1^ in the growing season versus 3–21 kg N ha^-1^ in the fallow season; Tables 4 and 5).

**Table 5:** Estimates of winter fallow season N mineralization and losses.

Estimated fallow season mineralization	Site 1		Site 2	
	(kg N ha^-1^)	SE	(kg N ha^-1^)	SE
N mineralized from crop residue, fallow season	16.9	1.3	21.0	2.1
Soil NO_3_-N, late spring^a^	20.3	0.7	42.1	0.5
Fallow season peat N mineralization^b^	3.4	0.3	21.0	2.2

^a^ Soil NO_3_-N accumulated prior to permanent flood establishment during the growing season is assumed to be lost to denitrification on flooding

^b^ Fallow season mineralization is the difference of NO_3_-N losses and N mineralized from residue

### Estimated soil C loss based on SOM-N mineralization

Total annual SOM-N mineralization, the sum of growing and fallow season mineralization, was 302 kg N ha^-1^ (Site 1) and 279 kg N ha^-1^ (Site 2) (Table 6,S7 Table). Based on the C:N ratio of the soil (14.4 at Site 1 and 14.8 at Site 2) and the total annual N mineralization, the total annual mass of soil C mineralized was 4345 kg C ha^-1^ (Site 1) and 4136 kg C ha^-1^ (Site 2) (Table 6,S7 Table). Accounting for the annual C input from crop residue following fallow season decomposition, the net C loss was 2473 kg C ha^-1^ (Site 1) and 2241 kg C ha^-1^ (Site 2) when NUE is assumed at 50% (Table 6,S7 Table). Soil C loss from this rice system is lower than reported values for other agricultural peatland systems (pasture and upland crops) in temperate regions which ranged from 3700–8600 kg C ha^-1^ yr^-1^ [17, 65, 66]. Similarly, modeled soil C losses from neighboring islands in the Delta where upland crops are grown ranged from 5000 to 15000 kg C ha^-1^ yr^-1^ [13]. The lower soil C losses estimated in this study are likely due to the seasonal flooding of the rice fields which reduces SOM oxidation in the system relative to conventional upland crops or pasture.

**Table 6:** Soil C loss estimate based on annual peat N mineralization and assuming a nitrogen uptake efficiency (NUE) of 50%.

Subsidence estimate	Site 1		Site 2	
	(kg ha^-1^)	SE	(kg ha^-1^)	SE
Annual peat N mineralization^a^	302	41	279	39
Annual C mineralized^b^	4345	596	4136	580
Annual C input from residue^c^	1872	137	1894	189
Net C loss	2473	612	2241	610

^a^ Sum of peat N mineralization from growing season (Table 4) and winter fallow season (Table 5)

^b^ Based on soil C:N ratio to 30 cm.

^c^ Based on residue samples at tillage

**Fig 3:**
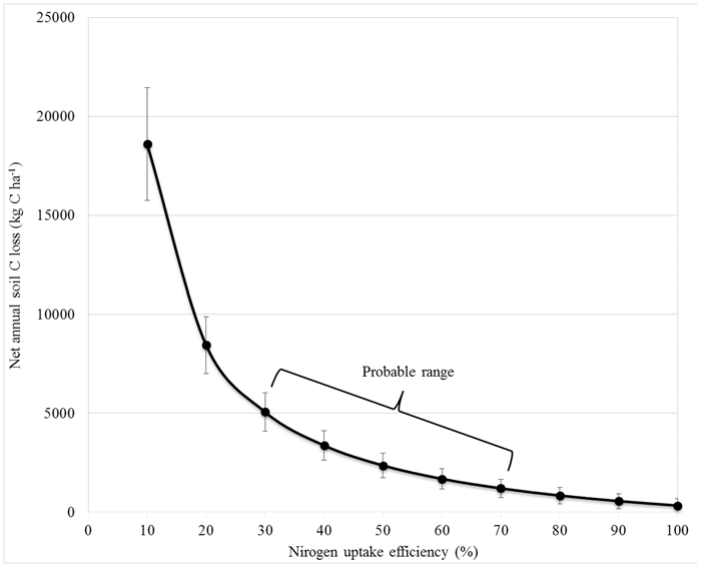
The effect of N uptake efficiency (NUE) assumptions on calculated soil C loss. Error bars are the standard error (n = 8) based on the final output from the soil C loss model, average of both sites, using conventional propagation of error calculations.

The N budget estimate of soil C loss is sensitive to NUE values used in the model (Fig 3). Lower values of NUE correspond to higher rates of soil C loss because an assumption of lower N uptake implies more N, and thus SOM, was mineralized relative to plant N uptake. In general, fertilizer NUE ranges between 30 to 70% in rice systems [40, 41, 42, 53]. In this study, we are considering a range of NUE slightly higher, from 50% to 70% because no accumulation of N was observed in the soil-water system during the growing season based on porewater and soil sampling (S5 and S6 Tables), suggesting mineralization was fairly synchronized with plant uptake. Also increases in NUE yield marginally smaller reductions in soil C loss, and standard errors are greatly diminished at 50% NUE and above, meaning that within this range the final net soil C loss estimate is less sensitive to the NUE assumption (Fig 3). There is reason to believe the NUE in this N fertilizer omission system is above the 50% threshold, as well-managed rice systems in California have reported NUE above 70% [30]. Considering the effect of our NUE assumption on estimated soil C loss, the probable range of 50–70% NUE would give a range of estimated net soil C loss from 1246–2473kg C ha^-1^ yr^-1^ (Site 1) and 1149–2241 kg C ha^-1^ yr^-1^ (Site 2).

Using the calculated range of net soil C loss from this study, it is possible to estimate the corresponding soil subsidence under rice production at this site. Because of the limited resolution of soil bulk density measurements in this study, the correlation between soil C loss and subsidence is dependent on an assumption about the fraction of subsidence due directly to C loss versus the effects of compaction and consolidation of the remaining mineral soil material. Studies in the California Delta have suggested that while C loss and subsidence rates have both been declining over time relative to the rapid losses after initial drainage, the relative importance of SOM mineralization losses has been increasing [13]. Deverel and Leighton (2010) estimated that 67% of subsidence since 1995 could be attributed to SOM losses on a neighboring Delta island [13], a value which agrees with work from other drained peatlands showing an increase in the relative importance of mineralization over time [67]. Using the SOM percent C (0.58 g C g^-1^ SOM) and soil bulk density in the upper 30 cm from our study sites on Twitchell Island (0.555 g cm^-3^ at Site 1 and 0.782 g cm^-3^ at Site 2), and the estimated fraction of subsidence due to SOM losses reported in Deverel and Leighton (2010) (f _min_, 0.67 [13]), it is possible to estimate a rate of annual subsidence. Assuming 50% NUE, we calculated net soil C loss equal to 2.473 * 10^-2^ g C cm^-2^ yr^-1^ (Site 1) and 2.241 * 10^-2^ g C cm^-2^ yr^-1^ (Site 2). Subsidence (cm yr^-1^) can then be estimated using the following equation:

$$Subsidence\;=\;[(net\;C\;loss^{}/\;SOM\;\%\;C\;)\;/\;bulk\;density]\;/\;f_{min}$$ (14)

The estimated annual subsidence by this method was 0.11 cm yr^-1^ at Site 1 and 0.07 cm yr^-1^ at Site 2. Some caution is needed, however, in interpreting these subsidence estimates because of the assumptions involved in calculating them, particularly the use of the soil bulk density and fraction of subsidence due to SOM loss. Yet these results are in close agreement with Hatala et al. (2012) who estimated 0.10–0.14 cm yr^-1^ subsidence loss at this site using eddy covariance tower measurements to develop and C budget [26]. Both these studies suggest that rice systems reduce subsidence relative to regional averages of 1–3 cm yr^-1^ [13], and rates measured in a maize field at the same site of 2.5 cm yr^-1^ [26]. Our analysis also indicates that after the first four years since conversion to rice agriculture, seasonal flooding is however not adequate to achieve soil gains similar to those observed in constructed wetlands [68, 69].

Measuring subsidence directly in a dynamic, intensely managed agricultural system is difficult, and thus all methods provide only estimates. Methods using extensometers to estimate the surface elevation relative to fixed anchors [c.f. 16] require specialized equipment and many years to examine a trend in the data. Methods using GHG flux measurements [c.f. 26] are costly and labor intensive. In this context, using this N budget approach may be preferable as it is less resource intensive and can easily be integrated with a farmer’s management practices on a small portion of land, and further study that could refine bulk density and consolidation measurements including an assessment of temporal change in these factors would be valuable to more directly link SOM-N mineralization estimates with ongoing subsidence in the field.

## Conclusions

Evidence from around the world suggests that our ability to manage SOM and reduce subsidence and C loss in peatlands under agricultural production is limited, generally resulting in peatland degradation. Based on the N budgeting approach in this study, transitioning high organic matter soils to flooded rice cultivation indicated a potential reduction in soil C loss and subsidence rates compared to traditional aerobic cropping; however, our calculations indicate that soil C loss continued at an estimated rate of 1149–2473 kg C ha^-1^ yr^-1^. Our results indicate that the N budget approach used in this study may be added to the repertoire of tools used to estimate subsidence and C loss, with opportunities for further refinement of model inputs and uncertainty. This study illustrates the complexity of mineralization and subsidence processes in seasonally flooded peatland soils. Further study is needed to assess sustainable long-term management solutions for these sensitive ecosystems.

## Supporting Information

S1 Table Surface irrigation water N content during the 2012 growing season. Sampled from irrigation inlet at each site while irrigation was on.
(DOCX)

S2 Table Aboveground biomass N uptake in 2012 mesocosm experiment. (DOCX)

S3 Table Groundwater N content from HydroFocus, Inc., well sampling, 7–8 August 2012. (DOCX)

S4 Table Overwinter rice residue decomposition, 2011–2012, Site 1. (DOCX)

S5 Table Soil N content during the 2012 growing season, sampled from N fertilizer omission plots. (DOCX)

S6 Table Soil porewater N content during the 2012 growing season, averages of four replicates. per site sampled from Rhizon-MOM porewater samplers installed in the mesocosm experiment.
(DOCX)

S7 Table Complete N budget calculator, 2011–2012. (DOCX)
