# Supplementary material for: Estimating Annual Soil Carbon Loss in Agricultural Peatland Soils Using a Nitrogen Budget Approach
Source: PLoS One. 2015 Mar 30;10(3):e0121432. doi: 10.1371/journal.pone.0121432 (PMC4379157; doi:10.1371/journal.pone.0121432)
Supplement: S1 Table — Sampled from irrigation inlet at each site while irrigation was on. (DOCX) [file pone.0121432.s001.docx]

|  | NH_4_-N | | NO_3_-N | |
| --- | --- | --- | --- | --- |
| Sample date | Site 1  (mg L^-1^) | Site 2  (mg L^-1^) | Site 1  (mg L^-1^) | Site 2  (mg L^-1^) |
| 7.22 | 0.00 | 0.00 | 0.15 | 0.13 |
| 8.4 | 0.67 | 0.62 | 0.00 | 0.00 |
| 8.14 | 0.69 | 0.72 | 0.24 | 0.29 |
| 8.31 | 0.08 | 0.09 | 0.03 | 0.09 |
