# Supplementary material for: Estimating Annual Soil Carbon Loss in Agricultural Peatland Soils Using a Nitrogen Budget Approach
Source: PLoS One. 2015 Mar 30;10(3):e0121432. doi: 10.1371/journal.pone.0121432 (PMC4379157; doi:10.1371/journal.pone.0121432)
Supplement: S2 Table — (DOCX) [file pone.0121432.s002.docx]

|  | Aboveground biomass N uptake | | | |
| --- | --- | --- | --- | --- |
|  | Site 1 | | Site 2 | |
| Treatment | N uptake  (kg N ha^‑1^) | SE | N uptake  (kg N ha^‑1^) | SE |
| + Water | 188.3 | 10.9 | 122.3 | 8.3 |
| - Water | 126.5 | 4.4 | 82.7 | 3.3 |
