# Supplementary material for: Estimating Annual Soil Carbon Loss in Agricultural Peatland Soils Using a Nitrogen Budget Approach
Source: PLoS One. 2015 Mar 30;10(3):e0121432. doi: 10.1371/journal.pone.0121432 (PMC4379157; doi:10.1371/journal.pone.0121432)
Supplement: S3 Table — (DOCX) [file pone.0121432.s003.docx]

| Site 1 | | | Site 2 | | |
| --- | --- | --- | --- | --- | --- |
| Sample depth (m) | NH_4_-N  (mg L^-1^) | NO_3_-N  (mg L^-1^) | Sample depth (m) | NH_4_-N  (mg L^-1^) | NO_3_-N  (mg L^-1^) |
| 1.5 | 6.2 | 0.0 | 1.8 | 3.2 | 0.0 |
| 3.0 | 7.4 | 0.0 | 2.3 | 2.3 | 0.0 |
| 5.2 | 4.9 | 0.0 | 3.3 | 18.0 | 0.0 |
|  |  |  | 3.7 | 17.0 | 0.0 |
