# Supplementary material for: Estimating Annual Soil Carbon Loss in Agricultural Peatland Soils Using a Nitrogen Budget Approach
Source: PLoS One. 2015 Mar 30;10(3):e0121432. doi: 10.1371/journal.pone.0121432 (PMC4379157; doi:10.1371/journal.pone.0121432)
Supplement: S4 Table — (DOCX) [file pone.0121432.s004.docx]

|  | Crop residue | SE |
| --- | --- | --- |
| Residue at harvest, fall 2011 (kg ha^-1^) | 9282 | 195 |
| Residue at tillage, spring 2012(kg ha^-1^) | 5060 | 370 |
| Percent residue decomposed overwinter fallow (%) | 45.5 |  |
