# Supplementary material for: Estimating Annual Soil Carbon Loss in Agricultural Peatland Soils Using a Nitrogen Budget Approach
Source: PLoS One. 2015 Mar 30;10(3):e0121432. doi: 10.1371/journal.pone.0121432 (PMC4379157; doi:10.1371/journal.pone.0121432)
Supplement: S5 Table — (DOCX) [file pone.0121432.s005.docx]

|  | Site 1 | | | | Site 2 | | | |
| --- | --- | --- | --- | --- | --- | --- | --- | --- |
|  | NH_4_-N  (mg L^-1^) | | NO_3_-N  (mg L^-1^) | | NH_4_-N  (mg L^-1^) | | NO_3_-N  (mg L^-1^) | |
| Cropping phase | Ave | SE | Ave | SE | Ave | SE | Ave | SE |
| Before planting | 7.83 | 1.15 | 35.86 | 1.06 | 3.23 | 0.15 | 12.41 | 0.47 |
| Before flooding | 1.62 | 0.09 | 20.35 | 1.01 | 2.50 | 0.18 | 42.09 | 0.18 |
| Late booting | 2.78 | 0.28 | 0.00 | 0.00 | 2.23 | 0.07 | 0.00 | 0.00 |
| Harvest | 0.85 | 0.08 | 0.83 | 0.18 | 1.11 | 0.11 | 1.60 | 0.21 |
| Before winter flooding | 0.94 | 0.07 | 4.33 | 0.57 | 1.42 | 0.14 | 1.19 | 0.21 |
