# Supplementary material for: Estimating Annual Soil Carbon Loss in Agricultural Peatland Soils Using a Nitrogen Budget Approach
Source: PLoS One. 2015 Mar 30;10(3):e0121432. doi: 10.1371/journal.pone.0121432 (PMC4379157; doi:10.1371/journal.pone.0121432)
Supplement: S6 Table — per site sampled from Rhizon-MOM porewater samplers installed in the mesocosm experiment. (DOCX) [file pone.0121432.s006.docx]

|  |  | NH_4_-N | | NO_3_-N | |
| --- | --- | --- | --- | --- | --- |
| Sample date | Treatment | Site 1  (mg L^-1^) | Site 2  (mg L^-1^) | Site 1  (mg L^-1^) | Site 2  (mg L^-1^) |
| 6.26 | + Water | 0.55 | 0.20 | 1.00 | 5.33 |
|  | - Water | 0.17 | 0.08 | 10.1 | 8.51 |
| 7.22 | + Water | 0.19 | 0.11 | 0.00 | 0.00 |
|  | - Water | 0.20 | 0.09 | 0.00 | 0.00 |
| 8.4 | + Water | 0.16 | 0.12 | 0.00 | 0.00 |
|  | - Water | 0.14 | 0.11 | 0.00 | 0.00 |
| 8.14 | + Water | 0.16 | 0.16 | 0.00 | 0.00 |
|  | - Water | 0.16 | 0.11 | 0.00 | 0.00 |
| 8.31 | + Water | 0.10 | 0.10 | 0.00 | 0.00 |
|  | - Water | 0.11 | 0.06 | 0.00 | 0.00 |
| 9.21 | + Water | 0.07 | 0.10 | 0.00 | 0.00 |
|  | - Water | 0.11 | 0.07 | 0.00 | 0.00 |
| 10.2 | + Water | 0.08 | 0.12 | 0.00 | 0.00 |
|  | - Water | 0.15 | 0.08 | 0.00 | 0.00 |
