# Supplementary material for: Estimating Annual Soil Carbon Loss in Agricultural Peatland Soils Using a Nitrogen Budget Approach
Source: PLoS One. 2015 Mar 30;10(3):e0121432. doi: 10.1371/journal.pone.0121432 (PMC4379157; doi:10.1371/journal.pone.0121432)
Supplement: S7 Table — (DOCX) [file pone.0121432.s007.docx]

| Twitchell 2012 N Budget Summary |  |  |  |  |  |  |
| --- | --- | --- | --- | --- | --- | --- |
| Fall 2011 - Fall 2012 | Site 1 | | | Site 2 | | |
|  | Calculator | SE | Relative error | Calculator | SE | Relative error |
| Total N uptake (kg N ha^-1^) | 185 | 10.0 | 0.05 | 167 | 7.7 | 0.05 |
|  |  |  |  |  |  |  |
| N derived from: |  |  |  |  |  |  |
| N deposition (wet and dry) (kg N ha^-1^) | 6 |  |  | 6 |  |  |
| BNF (kg N ha^-1^) | 25 |  |  | 25 |  |  |
| Straw (kg N ha^-1^) | 1.9 | 0.20 | 0.11 | 3.8 | 1.10 | 0.29 |
| Surface water (kg N ha^-1^) | 2.9 | 1.30 | 0.45 | 3.0 | 1.30 | 0.43 |
| Subsurface water (kg N ha^-1^) **assumed from peat | 61.7 | 9.0 | 0.15 | 39.6 | 6.70 | 0.17 |
| N accounted for by direct measure (kg N ha^-1^) | 66.5 | 9.1 | 0.14 | 46.4 | 6.91 | 0.15 |
| Surface peat mineralization 0-30cm (kg N ha^-1^) | 87.5 | 13.5 | 0.15 | 89.6 | 10.3 | 0.12 |
| Total peat (surface + subsurface) | 149.2 | 16.2 | 0.11 | 129.2 | 12.3 | 0.10 |
| Assumed N uptake efficicency | 0.50 |  |  | 0.50 |  |  |
| Growing season Peat N mineralization accounting for NUE (kg N ha^-1^) | 298.4 | 32.5 | 0.11 | 258.3 | 24.7 | 0.10 |
|  |  |  |  |  |  |  |
| Crop residue at harvest (kg N ha^-1^) | 9282 | 195.0 | 0.02 | 9282 | 195.0 | 0.02 |
| Crop residue % N at harvest | 0.007 |  |  | 0.007 |  |  |
| N in crop residue at harvest (kg N ha^-1^) | 65 | 1.4 | 0.02 | 65 | 1.4 | 0.02 |
| Crop residue remianing at tillage (kg N ha^-1^) | 5060 | 370.0 | 0.07 | 5120 | 510.0 | 0.10 |
| Crop residue % N at tillage | 0.0095 |  |  | 0.0086 |  |  |
| N in crop residue at tillage (kg N ha^-1^) | 48 | 3.5 | 0.07 | 44 | 4.4 | 0.10 |
| Total N mineralized from straw harvest-tillage (kg N ha^-1^) | 16.9 | 1.3 | 0.08 | 21.0 | 2.1 | 0.10 |
|  |  |  |  |  |  |  |
| Soil NO3-N at planting 0-15cm (kg N ha^-1^) | 35.9 | 1.1 | 0.03 | 12.4 | 0.5 | 0.04 |
| Soil NO3-N at permanent flood 0-15cm (kg N ha^-1^) | 20.3 | 0.7 | 0.03 | 42.1 | 0.5 | 0.01 |
| Losses assuming complete denitrification upon flooding (kg N ha^-1^) | 20.3 | 0.7 | 0.03 | 42.1 | 0.5 | 0.01 |
| Overwinter peat N mineralization (NO3 - overwinter straw mineralization) (kg N ha^-1^) | 3.4 | 0.3 | 0.08 | 21 | 2.2 | 0.10 |
|  |  |  |  |  |  |  |
| Total peat N mineralization (kg N ha^-1^) | 301.76 | 41.4 | 0.14 | 279.4 | 39.2 | 0.14 |
| C:N of bulk soil 0-30cm | 14.4 | 0.05 | 0.00 | 14.8 | 0.09 | 0.01 |
| Total C mineralized based on C:N ratio (kg C ha^-1^) | 4345.4 | 596 | 0.14 | 4136 | 580.1 | 0.14 |
| Total C input from straw at tillage (kg C ha^-1^) | 1872.2 | 137 | 0.07 | 1894 | 189.0 | 0.10 |
| Net soil C loss (kg C ha^-1^) | 2473.2 | 612 | 0.25 | 2241 | 610.1 | 0.27 |
